# Dynamic exacerbation in inflammation and oxidative stress during the formation of peritendinous adhesion resulted from acute tendon injury

**DOI:** 10.1186/s13018-021-02445-y

**Published:** 2021-05-05

**Authors:** Pengfei Li, Haiying Zhou, Tian Tu, Hui Lu

**Affiliations:** 1 Department of Plastic and Aesthetic Center, The First Affiliated Hospital, College of Medicine, Zhejiang University, #79 Qingchun Road, Hangzhou, 310003 Zhejiang Province China; 2 Department of Orthopedics, The First Affiliated Hospital, College of Medicine, Zhejiang University, #79 Qingchun Road, Hangzhou, 310003 Zhejiang Province China

**Keywords:** Peritendinous adhesion, Acute tendon injury, Tendon surgery, Reactive oxygen species, Inflammation, Dynamic exacerbation

## Abstract

**Background:**

Peritendinous adhesion is among the common complications after tendon injury. Numerous studies have been carried out to prevent its formation, including modifications of surgical procedures, postoperative cares, application of medicines, etc. This study dynamically monitored fluctuations of inflammation, state of oxidative stress, and histopathologic changes around injured tendon to provide theoretical basis for further exploration in mechanisms of peritendinous adhesion formation.

**Methods:**

Eighteen mature Sprague-Dawley male rats were randomly allocated into 6 equal groups. Compared with control and sham group, every rat’s right hind Achilles tendon in experimental groups was cut and repaired by the modified Kessler technique. Besides control and sham group, samples of tendon margin and serum were collected at different time points after the surgery. Content of TNF-α, IL-1β, and TGF-β were assayed in harvested serum. Reactive oxygen species (ROS) were detected, expression levels of related genes (IL-1β, IL-6, SOD1, SOD2, COL1, HIF1A) were quantified by qPCR, and various histopathological evaluations were performed.

**Results:**

Indicators (TNF-α, IL-1β, TGF-β1, ROS) were noticed to have a similar trend of significant rising 24 h after the surgery except TGF-β which was rising 72 h later. So were the expression trends of IL-1β, IL-6, SOD1, SOD2, and COL1. HIF1A, inversely correlated with SOD2, showed the progressive relief of regional tissue hypoxia. Histological evaluation showed the same tendency that fibrosis and inflammation were getting serious 48 h later after the surgery.

**Conclusions:**

Inflammation, oxidative stress in injured tendon resulted from acute trauma, would be getting intense in 24 h. Peritendinous adhesion emerges and aggravates after 48 h. Thus, prompt efficient measures are advised to be taken after the injury as soon as possible.

## Background

Peritendinous adhesion is one of the most common complications following tendon injuries [1]. It prevents tendon excursion during the healing process, and eventually cause the reduction of joint motion and result in poor function [2, 3]. Functional disturbances prolong rehabilitation, give rise to psycho-socioeconomic problems, and require reoperation [1, 4, 5]. Previously numerous methods have been carried out to prevent adhesion formation, including modifications of surgical techniques, use of mechanical barriers, systemic or local application of medications, and postoperative physical therapy [1, 2, 5–12]. Though most of them got positive results, the specific mechanisms underneath remain poorly explored. It is believed that adhesion would decrease and joint gliding be facilitated if peritendinous inflammation could be prevented in the repair period [5, 13, 14]. What is more, hypoxia-induced oxidative stress following tissue injury might be responsible for starting the process of fibrosis and leading to unnecessary tissue adhesion [15–17]. Though many researchers have explored about reactive oxygen species (ROS) [17, 18], there is barely any exploration connected them with trauma, especially tendon injuries.

In order to find the inner relationship of inflammation, oxidative stress and fibrosis of injured tendon, we dynamically monitored fluctuations of inflammatory state, reactive oxygen species, and histopathologic changes around injured tendon in the hope of providing theoretical basis for further exploration in the mechanisms of peritendinous adhesion formation and future treatment application, such as precise drug delivery.

## Materials and methods

### Rat Achilles tendon injury model

All animal experiments were performed according to the guidelines outlined by the National Technical Committee on Laboratory Animal Science of the Standardization Administration of China and approved by the Animal Welfare and Research Ethics Committee in the First Affiliated Hospital, College of Medicine, Zhejiang University (reference number:2019(990)).

**Fig. 1:**
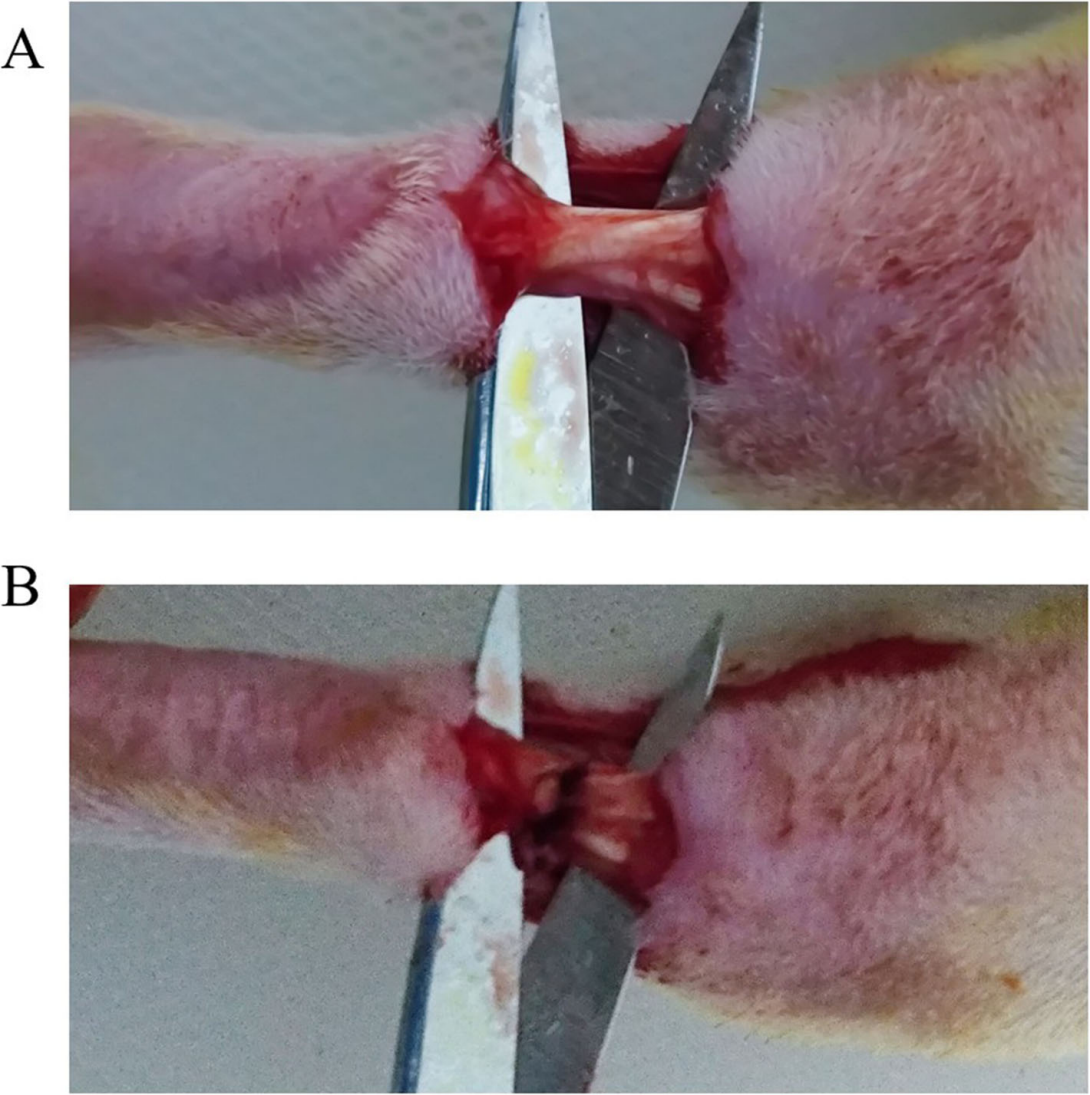
**a** Achilles tendon was carefully dissected. **b** The tendon was restructured using modified Kessler’s method after sharply severed

Eight-week-old Sprague-Dawley male rats were housed with freely available commercial rat chow and tap water and maintained on a 12-h light/dark cycle. They were raised to acclimate for 2 weeks prior to surgery. Anesthesia was implemented with intraperitoneal injection of 10% chloral hydrate (0.3 ml/100 g) after 12 h of fasting. Lying supine, all limbs were fixed except the right hind ones which were reserved for anastomosis of Achilles tendon. Mid-lateral incision of 3 cm was made in the forelimb to expose the Achilles tendon. Then, sharply sever the tendon obliquely in the middle and anastomose tendon ends using modified Kessler’s method with 5-0 Ti-Cron non-absorbable sutures (COVIDIEN, Langhorne, PA, USA). Finally, skin was closed with 5-0 Surgipro sutures (COVIDIEN, Langhorne, PA, USA) [11]. All surgical procedures were performed by the same surgeon (Fig.1).

### Grouping and harvesting

Eighteen Sprague-Dawley (SD) male rats weighing around 350 g were equally allocated into 6 groups. They were treated as follows: group 1 (control), no intervention; group 2 (sham), the identical skin incisions were made and closed with the tendon intact; groups 3, 4, 5, and 6 (tendon surgery), all rats went through the Achilles tendon surgery.

The serum and samples of the tendon tissue in every group were arranged to be harvested as follows: group 1, same time with group 2; group 2, 72 h after the fake surgery; group 3, 6 h after the tendon surgery; group 4, 24 h after the tendon surgery; group 5, 48 h after the tendon surgery; group 6, 72 h after the tendon surgery.

### Gross observation and macroscopic evaluation of tendon adhesion

The mental state, activity, fur, weight, and wound healing of SD rats were dynamically observed. Before sacrificing the animals, the repaired Achilles tendon was thoroughly exposed and visually examined for its healing and signs of inflammation and granulation tissue hyperplasia. The criteria for evaluation of peritendinous adhesion described by Tang et al. [11, 19] were adopted in our measurement Table 1

**Table 1:** Criteria for histological evaluation of peritendinous adhesion

Points	Features of adhesion
	Quantity
0	No apparent adhesion
1	A number of scattered filaments
2	A large number of filaments
3	Countless filaments
	Quality
0	No apparent adhesions
1	Regular, elongated, fine, filamentous
2	Irregular, mixed, shortened, filamentous
3	Dense, not filaments
	Grading of adhesion
0	None
1–2	Slight
3–4	Moderate
5–6	Severe

### Inflammation evaluation

To evaluate the degree of inflammation the injuries caused, content of TNF-α, IL-1β, and TGF-β1 in the serum were chosen as the key indicators. Then content of the indicators was detected by means of enzyme-linked immunosorbent assay (ELISA).

### Oxidative stress

The reactive oxygen species in the collected samples of each group were detected to measure the level of oxidative stress according to the instructions of the assay kit for ROS (chemical fluorescence method). The tendon samples were cut into pieces of 1 mm^3^, digested for 2 h with collagenase II at 37 °C and cell pellet was collected after centrifugation for 5 min at 1500 rpm. Then, the collected cell pellet was added 1mL of Dichloro-dihydro-fluorescein diacetate (DCFH-DA) which was diluted with serum-free medium to a final concentration of 10 μmol/L, incubated for 30 min at 37 °C and washed three times with serum-free medium. Before transferring to a 96-well plate, cells were centrifuged for 5 min at 1500 rpm and resuspended in phosphate-buffered saline (PBS). Finally, the value of each well was calculated by the fluorescence unit determined by a microplate reader at 488/525.

### RNA isolation and qPCR

RNA was extracted and purified from collected tendon tissues according to the manufacturer’s instructions using EZ-press RNA purification kit (EZbioscience, MN, USA) and quantified through a spectrophotometer (Molecular Devices, CA, USA). cDNA was prepared by reverse-transcribing total RNA with the Double-Strand cDNA Synthesis Kit (Takara). All gene transcripts were quantified by Power SYBR® Green PCR Master Mix (Takara) on the ABI StepOnePlus System (Applied Biosystems, Warrington, UK). The primer sequences were shown in Table 2. Expression levels were calculated using the relative quantitation method and gene expression was normalized to rat housekeeping gene *GAPDH* as previously reported [20].

**Table 2:** Rat primers used in quantitative PCR analysis

Gene	Primer sequence(5′-3′)
IL-1β	Sense: GGGATGATGACGACCTGCTA
	Antisense: TGTCGTTGCTTGTCTCTCCT
IL-6	Sense: CCACTGCCTTCCCTACTTCA
	Antisense:TTCTGACAGTGCATCATCGC
SOD1	Sense: CACTTCGAGCAGAAGGCAAG
	Antisense:CCAACATGCCTCTCTTCATC
SOD2	Sense: CACATTAACGCGCAGATCATG
	Antisense:CCTTAGGGCTCAGGTTTGTC
COL1	Sense: GGATCGACCCTAACCAAGGC
	Antisense:GATCGGAACCTTCGCTTCCA
HIF1A	Sense: TGCTTGGTGCTGATTTGTGA
	Antisense: GGTCAGATGATCAGAGTCCA
GAPDH	Sense:GAGCGAGATCCCGTCAAGATCAAA
	Antisense:CACAGTCTTCTGAGTGGCAGTGAT

### Histological evaluation

The repaired Achilles tendon tissues harvested were fixed in 4% buffered formalin (pH 7.4) and embedded in paraffin blocks. Serial sections of 4 um were sliced and stained with hematoxylin and eosin (H&E), and Masson staining. Preparations were viewed under an Olympus BX51 light microscope (Olympus; Tokyo, Japan) by two pathologists who were blinded to the design. Histologic grading methods were adopted to standardize the degree of adhesions and peritendinous inflammation among groups. The extent of adhesions was divided into four grades (1, no adhesions; 2, slight adhesions; 3, moderate adhesions; 4, severe adhesions) according to the criteria described by Tang et al. Peritendinous inflammatory reaction was classified into five grades (0, no reaction; 1, minimal leukocyte infiltration into fibro-osseous tendon sheath; 2, leukocyte infiltration in synovium and epitenon; 3, leukocyte infiltration in synovium and endotenon; 4, diffuse leukocyte infiltration) according to the criteria of Moran et al. [14].

For immunohistochemistry, sections were blocked with 5% bovine serum albumin for 1 h and incubated with collagen I (COL1) antibody (5 μg/mL; Abcam), collagen III (COL3) antibody (1:100; Affinity Biosciences, USA) at 4 °C overnight. After primary antibody incubation, samples were exposed to Rabbit HRP Polymer (Biocare Medical). The bound antibody complex was visualized with diaminobenzidine then counterstained with hematoxylin. Stained sections were prepared and viewed under light microscopy. Micrographs were collected using a camera-assisted microscope (Nikon Eclipse microscope, model E6000 with an Olympus camera, model DP79).

### Statistical analysis

All data were expressed as means + standard deviations. Student’s *t* tests were performed. Comparisons were made between surgery groups and control groups by analysis of variance. *P* values < 0.05 were considered significant.

## Results

### Macroscopic findings

All rats were observed to be in a healthy state and the skin wounds in every group recovered normally. No adhesion could be traced in the control and sham group. A gradual increasing trend of adhesion were seen from group 3 to group 6, while the biggest gap was observed between group 4 to group 5 which means the adhesion intensifies 48 h later after tendon repair. Table 3

**Table 3:** Grading results of macroscopic evaluation

	Group 1 (control)	Group 2 (sham)	Group 3 (6 h after surgery)	Group 4 (24 h after surgery)	Group 5 (48 h after surgery)	Group 6 (72 h after surgery)
Grading	0	0	1	2	3	6

### Inflammation

**Fig. 2:**
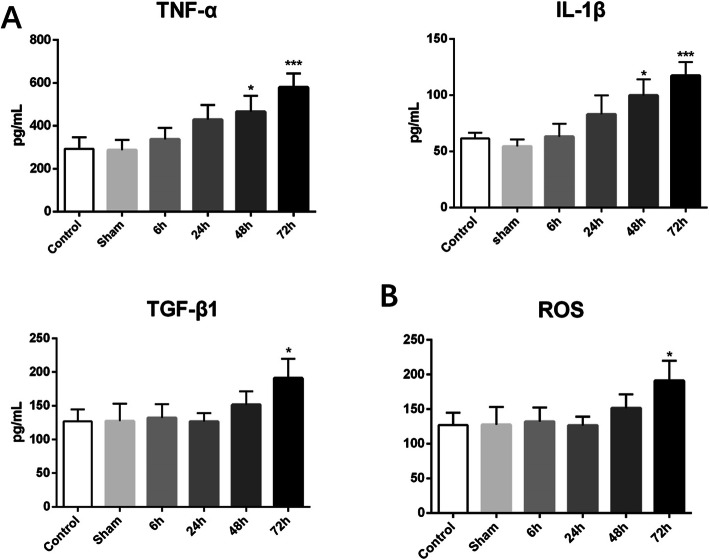
**a** Respective trends of TNF-α, IL-1β, and TGF-β1 in the serum with time. **b** Fluctuation of the serous content of ROS after tendon surgery ( * means *P* < 0.05 when compared with control or group, ***P < 0.001)

All three inflammatory indicators followed a similar trend of rising in tendon surgery group as time passed. Both TNF-α and IL-1β rose significantly 24 h later after the tendon repair surgery compared with the control and sham group (*P* < 0.05), while the extent of TGF-β1 statistically went up in 72 h after tendon surgery in the comparison with control and sham group (*P* < 0.05) (Fig. 2).

### Oxidative stress

The reactive oxygen species (ROS) which was measured to indicate tissue oxidative stress ascended significantly 48 h later after the tendon surgery compared with the control and sham group (*P* < 0.05) (Fig. 2).

### Uprising trends of mRNA expression

**Fig. 3:**
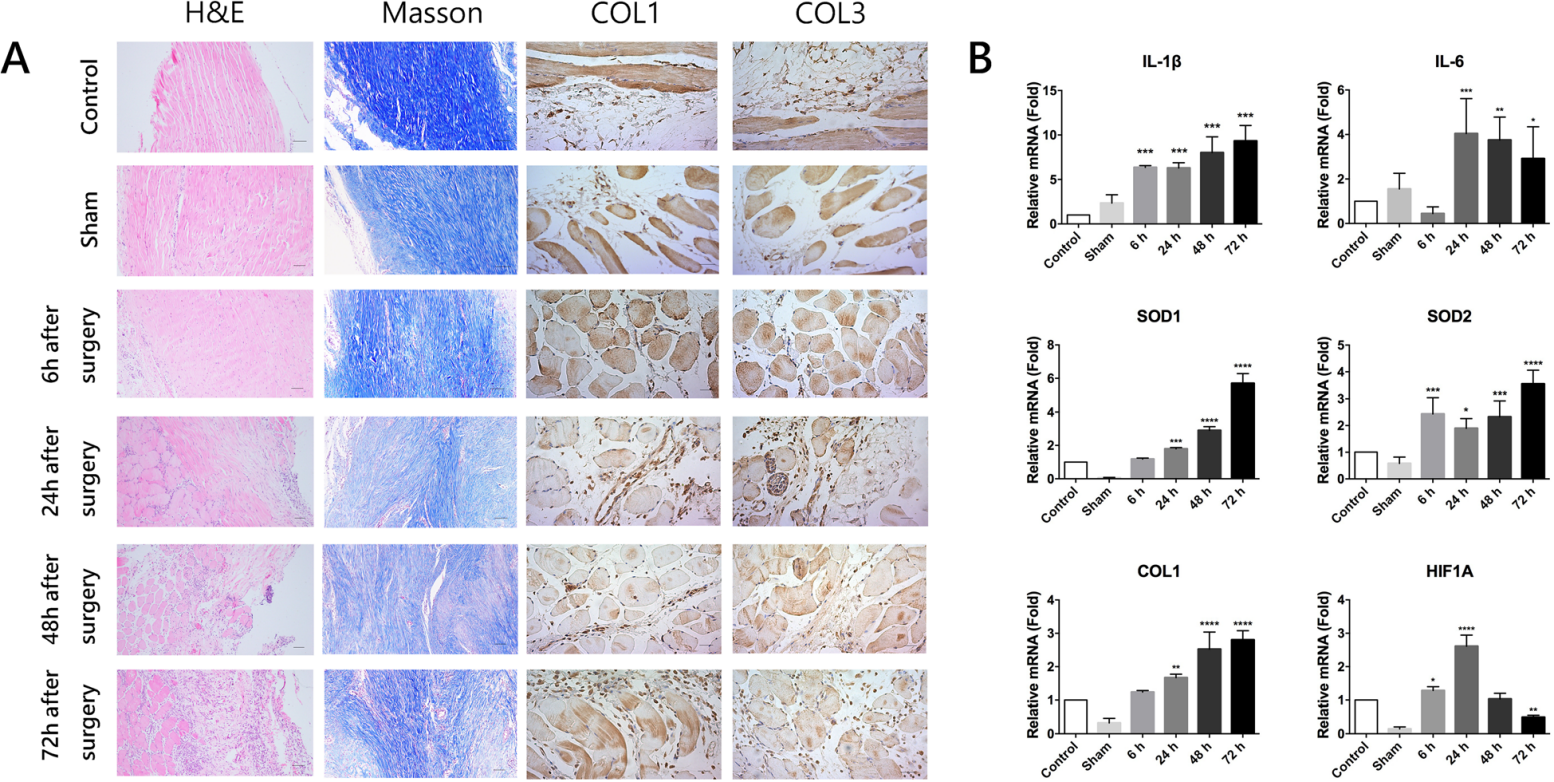
**a** Typical representation of histomorphologic findings; magnification, H&E and Masson, × 100; COL1 and COL3, × 200; bar = 200 μm. **b** Expression levels of diverse related genes during the process of recovery. Error bars indicated standard errors. (* means *P* < 0.05 when compared with control or group, **P < 0.01, ***P <0.001, ****P < 0.0001)

qPCR analysis revealed a clear rising trend in inflammation, oxidative stress and fibrosis formation after tendon surgery in the first 72 h. The expression of Interleukin-1β (IL-1β) and superoxide dismutase 2 (SOD2) in the experimental groups (groups 3, 4, 5, and 6) were significantly higher than that in the control and sham group (*P* < 0.05). Similar trend was seen in the expression of Interleukin-6 (IL-6) after 24 h. For superoxide dismutase 1 (SOD1) and collagen 1 (COL1), though the expression in experimental groups (groups 4, 5, and 6) were statistically higher than those of the control and sham group (*P* < 0.01), the uprising was gradual and accelerating after 48 h. The expression of hypoxia-inducible factor 1-alpha (HIF1A) was inversely proportional with SOD2 which indicated the dynamic situation of hypoxia and self-protection mechanisms by responsively raising SOD2. These results indicated that inflammation, oxidative stress, and fibrosis formation all gradually intensified in the very beginning. Thereinto, SOD1 and COL1, representing oxidative stress in cytoplasm and fibrosis formation, significantly ascended after 48 h (Fig. 3).

### Histological analysis

Histological results gathered from every group are shown in Fig. 3. Both H&E, Masson, and immunohistochemistry staining of COL1 and COL3 showed similar trends in histomorphology. No obvious peritendinous adhesion was observed in the control and sham group. The space around collagen fibers and peritendinous tissues was loose and clear. Collagen fiber hyperplasia and fibroblast proliferation were seen gradually increased from group 3 to group 6. With time, inflammatory cells, vessels, and fibrous connective tissue infiltrated the tendon bundle. The thickest and firmest fibrous adhesion tissues developed at the repair sites in group 6. Semiquantitative scoring analysis showed a statistically significant rising in adhesion formation 48 h later after tendon repair surgery (*P* < 0.05).

## Discussion

Peritendinous adhesion is one of the most common complications occurred during the healing process of injured tendon [1, 21, 22]. It binds the flexor tendons, restricts tendon gliding, and consequently leads to poor joint function [23, 24]. Numerous attempts have been explored to prevent adhesion formation [2–5, 7–10, 13, 14, 21, 25]. For example, modified Kessler’s method, improvements in surgical technique, was proved to decrease the adhesion formation by 134% [1, 4]. Other explorations, including local application of hyaluronic acid, 5-FU [5], etc. [3, 10]. or physical barriers such as collagen membranes [26], PGA membranes [27], etc. [24] were proved to positively affect adhesion formation. However, the mechanisms underneath remain unclear.

ROS, by products of various enzymatic reactions, regulate cellular homeostasis and mediate both physiological and pathophysiological signal transduction [28]. They have been proved to be closely associated with metabolic regulation, including the emergence of atherosclerosis [29], diabetes [30], and stroke [31], and are mechanically known in response to inflammation [28, 32]. Thus, ROS was included in our study as a potential breach into the mechanisms of the repair of injured tendon.

Tendon injury means disruption to the original vasculature and the rapid influx of metabolically demanding cells in the following repair process of adhesion formation [16]. In other words, tissue hypoxia, inflammation, and oxidative stress are main vital issues needed to be handled to prevent fibrosis as soon as the injury occurs. From here, we designed the experiments to study the dynamic fluctuations and relationships within inflammation, oxidative stress, and adhesion formation. It is obviously seen from our results that the extent of TNF-α, IL-1β, and reactive oxygen species (ROS) in tendon surgery groups all rose up significantly 24 h later which demonstrated a close correlation between postoperative inflammation and oxidative stress. The excess TGF-β1 after 72 h, which could lead to chronic inflammation, fibrosis, and accumulation of extracellular matrix [33], undoubtedly played an important role in the formation of peritendinous adhesion. The expression levels of IL-1β, IL-6, SOD1, SOD2, and COL1 all significantly ascended with time which confirmed the rising trend of inflammation, oxidative stress, and fibrosis formation. Expression of IL-1β, IL-6, and SOD2, representing inflammation and oxidative stress in mitochondria, relatively rose in 24 h which suggested instant reaction in inflammation and oxidative stress. While SOD1 and COL1, symbols for oxidative stress in cytoplasm and fibrosis formation, significantly increased after 48 h indicating the expansion of oxidative stress and the intensification of fibrosis. Moreover, the expression level of HIF1A altered in an opposite trend when compared with SOD2, indicating the progressive relief of regional tissue hypoxia. As for the histological findings, inflammatory and fibrous infiltrations were gradually intensified in a time-dependent manner. However, serious peritendinous adhesion was seen 48 h later after tendon repair surgery which indicated fibrosis exacerbated after 48 h. Combining all the results, it was deduced that in the first 24 h after tendon injury, vasculature loss and abrupt rising of metabolically demanding cells led to local tissue hypoxia which triggered following inflammation and oxidative stress. Under the influence of high levels of TNF-α, IL-1β, and reactive oxygen species (ROS), peritendinous adhesion emerges and aggravates in 48 h. However, in this stage, the effects of TGF-β1 were relatively minor until 72 h. Though TNF-α, IL-1β, TGF-β1, and ROS were all getting intensified over time, TNF-α, IL-1β, and ROS were quick to react, but TGF-β1 mainly affected the fibrosis 48 h later after tendon injury. Based on our primary study on peritendinous adhesion formation, in the first inflammatory phase (24–48 h), cytokines like TNF-α, IL-1β, and reactive oxygen species were the main problems needed to be studied possibly through exploring the canonical NF-κB pathway [28, 32]. In the next phase of fibroblast proliferation (after 48 h), besides the inflammation and oxidative stress, TGF-β1 was participated in the accumulation of extracellular matrix and fibrosis through potential pathways including epithelial-mesenchymal transition, immunity, and stromal signaling, etc. [33]. In our previous researches in the mechanisms of tendon adhesion after injury, we found that over-expression of miR-29b could downregulate the expression of TGF-β1 and Smad3 which aggravate adhesion [12]. Plus, application of Tanshinone IIA can inhibit the expression of TGF-β1 and reduce collagen production in fibroblasts, thereby restraining tendon adhesions in rats [11]. Our study shined the directions in which the mechanisms of early stage peritendinous adhesion formation might lay. What is more, the late stage of tendon recovery in remolding phase is still waiting to be explored.

In the future, we can further explore the mechanisms of peritendinous adhesion by connecting the canonical NF- κB with TGF-β1/Smad3 pathway, monitoring downstream expressions after regulation of target genes, and verifying in vivo by constructing a target gene knockout animal model.

## Conclusion

Inflammation, oxidative stress in injured tendon resulted from acute trauma would be getting intense in 24 h. Peritendinous adhesion emerges and aggravates after 48 h. Thus, prompt efficient measures are advised to be taken after the injury as soon as possible.

## Data Availability

The datasets used and/or analyzed during the current study are available from the corresponding author on reasonable request.

## Abbreviations

ROS: Reactive oxygen species
ELISA: Enzyme-linked immunosorbent assay
TNF-α: Tumor necrosis factor alpha
IL-1β: Interleukin 1 beta
IL-6: Interleukin 6
TGF-β1: Transforming growth factor beta 1
COL: Collagen
SOD: Superoxide dismutase
HIF1A: Hypoxia-inducible factor 1-alpha
SD rats: Sprague-Dawley rats
PBS: Phosphate-buffered saline
DCFH-DA: Dichloro-dihydro-fluorescein diacetate
GAPDH: Glyceraldehyde-3-phosphate dehydrogenase
